# Vocal Fold Augmentation with Beta Glucan Hydrogel Cross-Linked by *γ* Irradiation for Enhanced Duration of Effect:* In Vivo* Animal Study

**DOI:** 10.1155/2015/592372

**Published:** 2015-12-13

**Authors:** Youn-Mook Lim, Bo Hae Kim, Hee-Bok Kim, EunJi Park, Seok-Won Park, Jong-Seok Park, Se In Choi, Tack-Kyun Kwon, Seong Keun Kwon

**Affiliations:** ^1^Research Division for Industry and Environment, Korea Atomic Energy Research Institute, 29 Geumgu-gil, Jeongeup 580-185, Republic of Korea; ^2^Department of Otorhinolaryngology-Head and Neck Surgery, Seoul National University Hospital, 101 Daehak-ro, Seoul 110-744, Republic of Korea; ^3^Department of Otorhinolaryngology-Head and Neck Surgery, Dongguk University Ilsan Hospital, 814 Siksa Dong, Goyang 410-773, Republic of Korea; ^4^QueGen Biotech, Jeongwang 1-dong, Siheung-si 429-931, Republic of Korea; ^5^Department of Otorhinolaryngology, College of Medicine, Seoul National University, 101 Daehak-ro, Seoul 110-744, Republic of Korea; ^6^Cancer Research Institute, 101 Daehak-ro, Seoul 110-744, Republic of Korea; ^7^Seoul National University Biomedical Research Institute, 101 Daehak-ro, Seoul 110-744, Republic of Korea

## Abstract

This study explored a novel strategy to restore the vocal gap by using cross-linked *β*-glucan hydrogel by *γ*-irradiation. An aqueous solution of 5 wt% *β*-glucan was prepared and cross-linked using ^60^Co *γ* irradiation. Ten nude mice were injected with 0.8 mL of irradiated *β*-glucan on the left back and the same volume of nonirradiated *β*-glucan on the right back for comparison. The mice were sacrificed at 1 and 2 weeks after injection and histological evaluations were performed. Irradiated *β*-glucan demonstrated a significantly larger volume than nonirradiated *β*-glucan in the back of nude mice with less inflammatory reaction. After unilateral recurrent laryngeal nerve section in New Zealand White rabbits, irradiated and nonirradiated *β*-glucan were injected into paralyzed vocal folds. Irradiated *β*-glucan remained at the paralyzed vocal fold without definite inflammatory signs on endoscopy. High-speed recordings of vocal fold vibration showed decreased vocal gap in irradiated group compared to nonirradiated group. Histologically, the laryngeal epithelium and lamina propria remained intact, without inflammatory cell infiltration. Our newly developed injection material, irradiated *β*-glucan, showed excellent biocompatibility and remained longer than nonirradiated *β*-glucan* in vivo*, suggesting irradiated hydrogels as a new therapeutic approach that may be useful for the long-term treatment of vocal fold palsy.

## 1. Introduction

In recent years, injection laryngoplasty has gained popularity for glottal insufficiency, because of its initial low cost, minimal invasiveness, and low morbidity, compared with laryngeal framework surgeries, such as thyroplasty type I and arytenoid adduction [1]. Various types of hydrogels and microspheres have been proposed and tested clinically for unilateral vocal fold paralysis [1, 2]. However, none has the ideal characteristics required for an injection laryngoplasty material. The ideal vocal fold injection material would be readily available with minimal preparation, inexpensive, inert, resistant to resorption, easy to use, nontoxic, and completely biocompatible [3].

Hydrogels remain for a limited period* in vivo*, so modification of hydrogels is needed to lengthen the residence of the injected material. Irradiation techniques have been used for the formation of hydrogels [4]. These techniques are convenient because the chemical and physical properties of materials can be readily shaped by the radiation dose. Such radiation-processing techniques modify the properties of the polymeric materials, making them biocompatible [5]. The effects of high-energy radiation on polymers are generally divided into main chain scission and cross-linking. Longer reaction times allow the formation of more complete polymer networks, which have special characteristics, such as increased gel flexibility, swelling capacity, and mechanical strength [6]. In particular, *γ*-rays do not need the addition of initiators or cross-linkers to start the process, unlike other methods [4]. Additionally, *γ*-ray irradiation is usually used in the synthesis and sterilization of polymeric materials, thus reducing costs and production times [7].

*β*-Glucans belong to a group of polysaccharides that are present in the cell walls of bacteria, fungi, including mushrooms, and cereals, such as barley and oats. They are considered biological response modifiers with immunomodulatory and beneficial health effects, including anticancer properties [8, 9]. They also offer other important therapeutic properties including antioxidant, cholesterol- and glucose-lowering, antihypertensive, anti-inflammatory, antiviral, and antimicrobial effects [10–12]. *β*-Glucans are FDA-approved as “generally recognized as safe” (GRAS) [13]. Most studies on extracts containing *β*-glucans have found that they are well-tolerated by humans and animals [14].

Even though various biocompatible materials were tested for vocal fold augmentation, all materials could be a cause of adverse effects such as foreign body reaction and interference of vocal fold vibration. In addition, migration of injected particle is also a well known problem of vocal fold injection [15].

As mentioned above, *β*-glucan is considered as biocompatible material for human. However, there is no study investigating the biocompatibility of irradiated *β*-glucan hydrogel in animal or human vocal folds.

In this study, additive-free *β*-glucan hydrogels with high cross-linking densities were prepared using radiation cross-linking with *γ*-rays. The objectives of the study were to evaluate the ability of irradiated *β*-glucan hydrogel to remain at the injection site longer than nonirradiated *β*-glucan hydrogel, restore vocal fold vibration, and avoid inflammatory reaction when injected at the paralyzed vocal fold.

## 2. Materials and Methods

### 2.1. Preparation of Irradiated *β*-Glucan Hydrogel

Large-scale production of *β*-1,6-branched-*β*-1,3-glucan using semicontinuous fermentation of QG143-1, a* Schizophyllum commune* strain, was performed and materials were supplied by QueGen Biotech Inc. (Siheung, Republic of Korea). All reagents were used without further purification. Distilled water (DW) was used as a solvent in the experiments.

**Figure 1:**
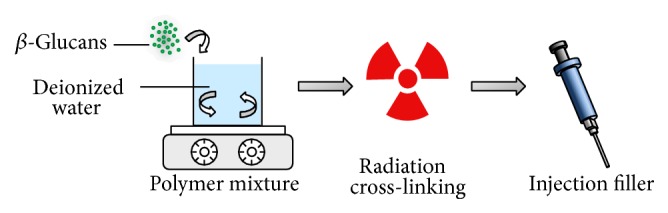
Schematic showing the fabrication process of *β*-glucan hydrogel by radiation cross-linking.

An aqueous solution of 5 wt%  *β*-glucan was prepared using DW. To obtain a homogenous solution, the solution was stirred and then kept on a hot plate at 50°C for 24 h. The solution was put into 1 mL sterilized syringes. The injection filler from the *β*-glucan solution was cross-linked with ^60^Co *γ*-ray irradiation doses of 5 kGy (dose rate: 5 kGy/h) at room temperature (Figure 1).

After fabrication, the same volumes of irradiated and nonirradiated *β-*glucan hydrogel were maintained in Dulbecco's modified Eagle's medium (DMEM) containing 1 g L^−1^ glucose and 1% penicillin/streptomycin (PS) and then incubated at 37°C under CO_2_ conditions for 1 month to examine the durability of the hydrogel* in vitro*.

### 2.2. Comparison of Volume Change and Tissue Responses* In Vivo*

Ten 6-week-old female athymic nude mice (BALB/c-nu/nu) were used to evaluate the volume change of injected *β*-glucan hydrogel and tissue responses to the irradiated *β*-glucan hydrogel in the body; these were compared with those of the nonirradiated hydrogel. Experiments were carried out in accordance with the guidelines of the Animal Research Committee, Dongguk University Ilsan Hospital. The protocol was approved by the Institutional Review Board of the Dongguk University Ilsan Hospital (the Institutional Care and Use Committee equivalent for Dongguk University Ilsan Hospital, Permit Number: 2013-176).

The back of each animal was washed and disinfected with 70% alcohol. The irradiated *β*-glucan was administered subcutaneously on the left side dorsum, and the same amount of nonirradiated *β*-glucan was injected on the right side dorsum. Each injection (0.8 mL) was performed with a syringe and a 25 G needle. At 1, 3, 7, 10, and 14 days after injection, volume changes were evaluated. The changes in injected volume were estimated by taking photographs and measuring the three dimensions (short/long diameter and height).

For a histological study, animals were euthanized by means of cervical dislocation at days 7 and 14 after implantation. The implants with surrounding tissue were removed and fixed with 4% formaldehyde for 24 h. The tissue blocks were cut into 5 *μ*m sections and stained with hematoxylin and eosin (H&E).

### 2.3. Recurrent Laryngeal Nerve Section for Unilateral Vocal Fold Palsy Animal Model

To prepare the unilateral vocal fold palsy animal model, 15 male New Zealand White rabbits (Koatech Laboratory Animal Company, Republic of Korea), weighing 2.6–3.0 kg, were used. Rabbits were anesthetized with a combination of zoletil (50 mg/kg) and xylazine (4.5 mg/kg), administrated intramuscularly. Surgery was performed as described previously [1]. Briefly, a vertical skin incision was made at the midline of the neck. The subcutaneous fat and the strap muscles were separated in the midline. The recurrent laryngeal nerve was identified by further dissection parallel to the inferior thyroid vessels. A cut 2 cm long was made in the left recurrent laryngeal nerve. The subcutaneous tissue and the skin were then replaced and sutured.

### 2.4. Implantation of Irradiated and Nonirradiated *β*-Glucan Hydrogel into the Paralyzed Vocal Fold

At 1 week after the recurrent laryngeal nerve sectioning, the irradiated *β*-glucan was injected into the paralyzed vocal folds of 20 (*n* = 15 for irradiated, *n* = 5 for nonirradiated rabbits), as described previously [1]. Each injection (100 *μ*L/rabbit) was made through a syringe with a 25 G spinal needle under the guidance of a 4.0 mm 30° rigid endoscope (Richards, Knittlingen, Germany). The injection needle was placed just lateral to the tip of the vocal process, so that the vocal process could rotate medially.

Postoperatively, the animals were observed for 2 h before being returned to their cages, where water and standard feed were available and clinical signs were monitored daily.

### 2.5. Laryngoscopic Evaluation

Immediately after the recurrent laryngeal nerve sectioning, a laryngeal endoscopic examination was performed using a 4.0 mm 30° rigid endoscope (Richards). Unilateral vocal fold immobility and vocal fold bowing were identified. Images of the vocal fold of each animal were taken with a digital camera (E4500, Nikon, Tokyo, Japan) attached to the rigid endoscope. At 1, 2, 4, 8, and 12 weeks after injection of the material, rabbits were anesthetized using the same method and the laryngeal endoscopic examination was repeated.

### 2.6. Recording of Induced Vocal Fold Vibration with High-Speed Camera and Functional Analysis

At 12 weeks after injection, five rabbits injected with irradiated *β*-glucan and five rabbits injected with nonirradiated *β*-glucan were sacrificed. Vocal fold vibration was examined using an excised laryngeal setup [1].

Recording of the rabbits' vocal fold vibration during induced phonation was accomplished with a MotionXtra NR4S2 high-speed video camera (DEL Imaging Systems, Cheshire, CT, USA). High-speed video data were recorded at 8,000 images/s and a spatial resolution of 256 horizontal × 512 vertical pixels. Illumination was provided with a 300-W xenon light.

From five cycles of vocal fold vibration, the five images with maximal glottal gap area within each cycle were chosen, and the glottal gap area was measured using Image J software (version 1.42; National Institutes of Health, Bethesda, MD, USA). The glottal gap area was compared between the irradiated and nonirradiated *β*-glucan groups by the Mann-Whitney test, using SPSS 21.0 (SPSS Inc., Chicago, IL). *P* values of less than 0.05 were considered statistically significant.

### 2.7. Laryngeal Histology and Volume Calculation

At 1 (*n* = 5), 4 (*n* = 5), and 12 (*n* = 5) weeks after injection of the irradiated *β*-glucan hydrogel, rabbits were euthanized by CO_2_ asphyxia under general anesthesia, and the larynges were removed by total laryngectomy. The specimens were fixed for 24 h in 10% formalin and sectioned serially in the axial plane from the false vocal fold to the subglottis. Histological sections (5 *μ*m thick) were stained with hematoxylin and eosin (H&E). The volume of remaining material was calculated by a method described previously [1]. At each follow-up, the dimensions of the remaining injected material were measured in pixels by using a microscopic computerized image analysis program (Leica Q win V3, Konvision Corporation, Seoul, Republic of Korea). The same procedure was repeated for ten sections that were 100 *μ*m apart. The volume of the remaining injected material was then estimated using the formula: 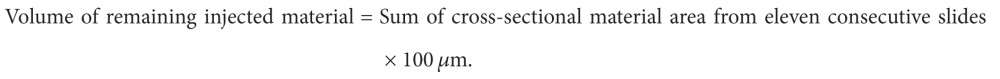
(1)


Volumes in each group were compared with the Kruskal-Wallis test, using the SPSS software (ver. 21.0). *P* values of less than 0.05 were considered to indicate statistical significance.

## 3. Results

All animals (mice and rabbits) survived without complications after subcutaneous injection and injection laryngoplasty. The irradiated *β*-glucan hydrogel maintained its shape in the media for a month, whereas the nonirradiated *β*-glucan hydrogel degraded completely (Figure S1; see Supplementary Material available on http://dx.doi.org/10.1155/2015/592372).

**Figure 2:**
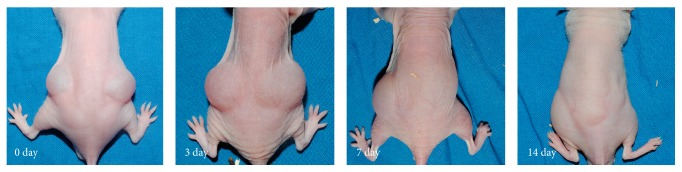
Serial findings of the mice after subcutaneous injection of irradiated (left side) and nonirradiated (right side) *β*-glucan. The volume of the irradiated hydrogel was maintained until 2 weeks whereas the nonirradiated hydrogel was absorbed completely; the photographs from the left to right were serially taken immediately after injection, and at 3 days, 7 days, and 2 weeks after injection.

**Figure 3:**
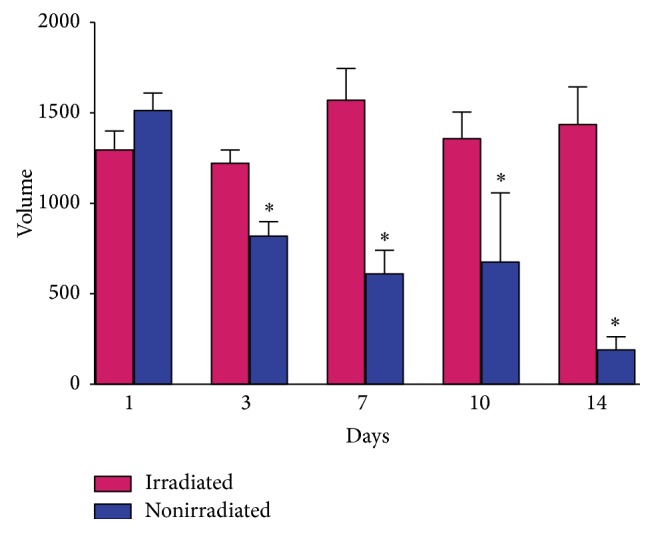
Comparison of the remaining volume of injected hydrogel. ^*∗*^ *P* < 0.05.

**Figure 4:**
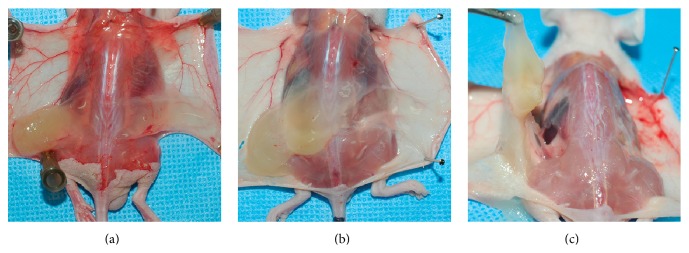
Gross findings after sacrificing the mice and subcutaneous flap elevation to show the residual hydrogel (irradiated hydrogel on the left side and nonirradiated hydrogel on the right side). The photographs were taken at (a) 1 week and (b) and (c) 2 weeks after injection. (a) At 1 week after injection, more irradiated hydrogel remained and it was more glutinous than the nonirradiated hydrogel. (b) At 2 weeks after injection, the nonirradiated hydrogel had almost disappeared whereas the irradiated hydrogel maintained almost the same volume. (c) The shape of the irradiated hydrogel was maintained and it did not fall apart even after elevation with forceps.

**Figure 5:**
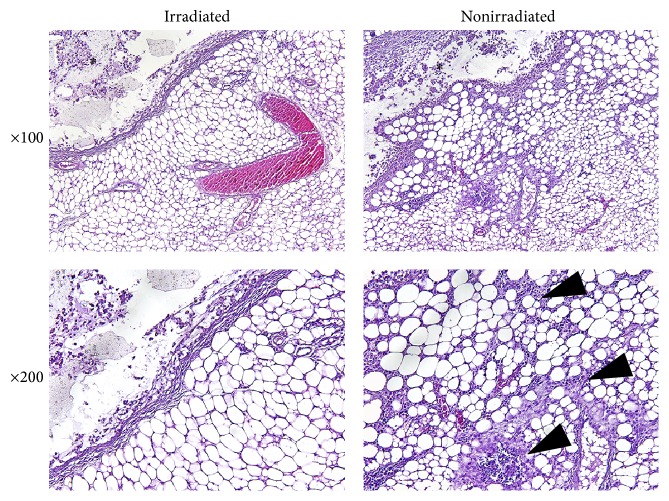
H&E staining of subcutaneous mouse tissue. The nonirradiated hydrogel caused more inflammatory reactions in the surrounding fat than did the irradiated hydrogel (black arrowhead: inflammatory cell infiltration into the fatty tissue; *∗*: the space in which the hydrogel was located).

**Figure 6:**
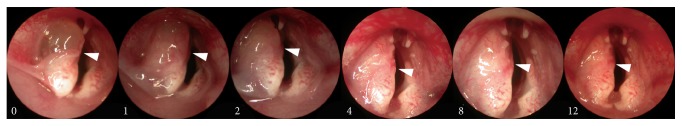
Serial endoscopic findings after injection of the paralyzed vocal fold with irradiated hydrogel. From the left, immediately after injection, and at 1, 2, 4, 8, and 12 weeks after injection. The volume of the injected materials decreased slowly on endoscopy over time, while the vocal processes of the injected vocal folds (white arrowheads) were continuously rotated medially.

After 2 weeks, much more of the irradiated *β*-glucan hydrogel than the nonirradiated hydrogel remained in the backs of the nude mice (Figure 2). The volume of irradiated hydrogel remaining was significantly larger than that of the nonirradiated hydrogel from the third day after injection (Figure 3). After sacrifice and cutaneous flap elevation at 1 week after injection, the irradiated hydrogel (left side of back) was much larger in volume and more glutinous than the nonirradiated hydrogel (Figure 4(a)). At 2 weeks after injection, the nonirradiated hydrogel had almost disappeared whereas the volume of the irradiated hydrogel was almost the same as when injected (Figure 4(b)). The irradiated hydrogel maintained its shape and did not fall apart even after elevation with forceps (Figure 4(c)). The nonirradiated hydrogel caused a more marked inflammatory reaction in the surrounding fat compared to what the irradiated hydrogel did (Figure 5). On endoscopic evaluation, the injected irradiated *β*-glucan hydrogel remained well in the paralyzed vocal fold for 12 weeks (Figure 6). There was no inflammatory response, such as granulation formation or hyperemia, at the vocal fold.

**Figure 7:**
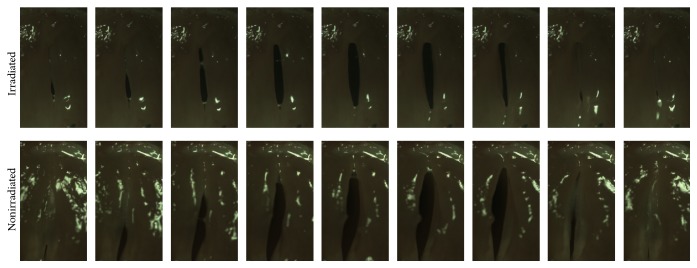
Serial vocal fold movements assessed using a high-speed camera. Vocal folds on both sides showed synchronous movements. Vocal fold gap was larger in nonirradiated group.

**Figure 8:**
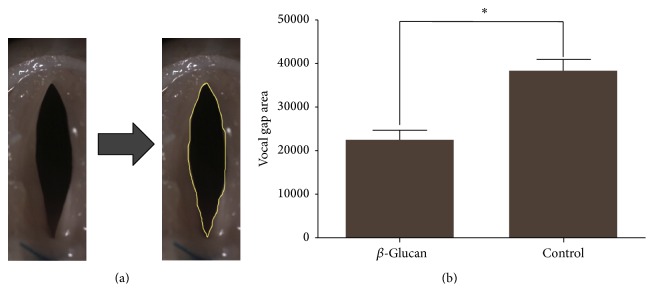
Analysis of vocal fold gap area from images with maximal glottal gap area within each cycle of vocal fold vibration. (a) Method to calculate the vocal gap. (b) Comparison of the vocal gap area measured 12 weeks after injection of irradiated or nonirradiated *β*-glucan. ^*∗*^ *P* < 0.05.

**Figure 9:**
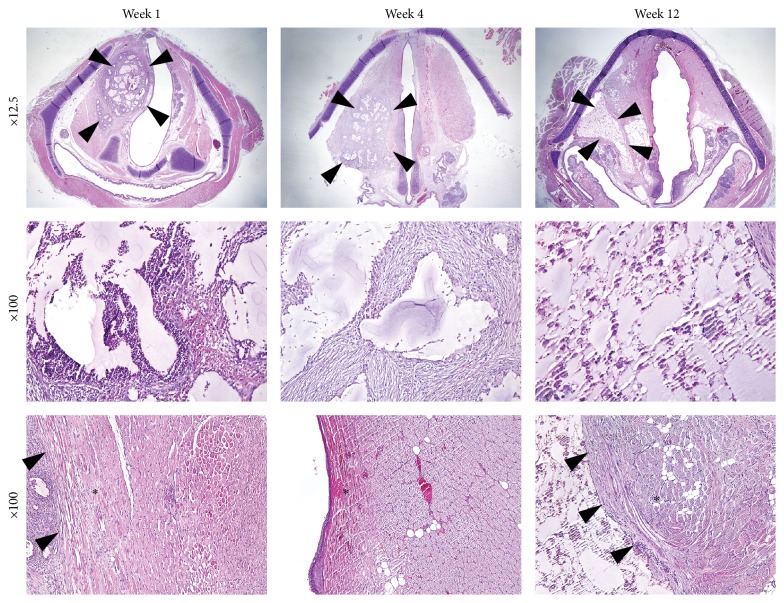
H&E staining of augmented vocal fold. Histological sections showed that injected *β*-glucan hydrogel remained until 12 weeks (border of the hydrogel: black arrowheads). At the first week, neutrophils, lymphocytes, and histiocytes were identified, suggesting a subacute inflammatory response. At the fourth week, neutrophils were rarely identified, while histiocytes and fibroblasts had increased in numbers. At week 12, lymphocytes, histiocytes, and giant cells were noted. No inflammatory reaction in the surrounding muscles or nearby epithelium was observed (asterisks).

**Figure 10:**
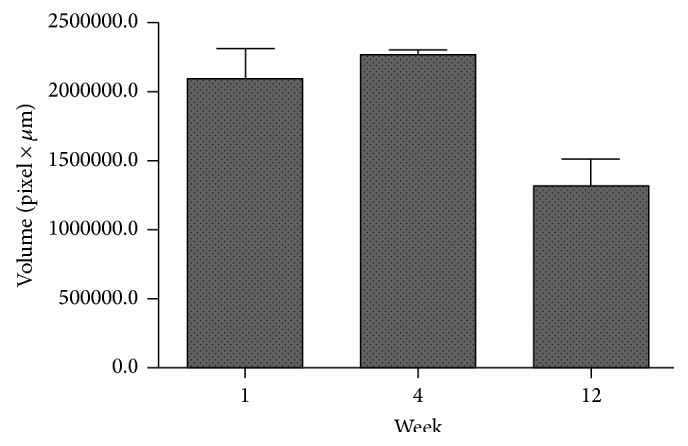
Comparison of the volume of the injected materials remaining at 1, 4, and 12 weeks after injection. The volume remaining did not differ significantly between the groups.

Serial photographs during one cycle of vocal fold vibration showed a much larger gap between the two vocal folds in the nonirradiated hydrogel group than in the irradiated hydrogel group (Figure 7). Vocal fold vibration was symmetric between right and left vocal fold in irradiated group, while the vocal fold vibration in nonirradiated group showed asymmetric movement and paradoxical motion (one vocal fold moving toward midline while the other vocal fold moving in the opposite direction). The vocal gap area was significantly smaller in the irradiated hydrogel group (Figure 8, *P* = 0.03). Histological analyses showed well-localized hydrogel in the larynx and did not reveal inflammatory tissue reactions in the epithelium or surrounding intralaryngeal muscles (Figure 9). The volume of hydrogel remaining tended to decrease with time, but not statistically significantly so (Figure 10, *P* = 0.140).

## 4. Discussion

In this study, we sought to assess a novel irradiated *β*-glucan hydrogel for augmentation of vocal folds that have lost glottal contact because of vocal fold palsy. The current study found that much more irradiated *β*-glucan hydrogel than nonirradiated hydrogel was retained at the injection site, without significant complications.

*β*-Glucan was chosen as the basis for this new material because it is already generally regarded as safe by the US Food & Drug Administration [13]; thus our modification had a high probability of being biocompatible. *β*-Glucan was chosen also because it has some anticancer properties. Vocal fold palsy sometimes results from direct invasion of the recurrent laryngeal nerve due to thyroid cancer or laryngeal cancer and there is the possibility of residual microscopic tumors near a paralyzed vocal fold at the time of injection laryngoplasty.

All *β*-glucans are glucose polymers linked by a (*β* 1–3) linear *β*-glycosidic chain core, and they differ in their lengths and branching patterns (Figure S2). The branches derived from the glycosidic chain core are variable and the two main groups of branching are (*β* 1–4) glycosidic chains, mainly in the *β*-glucans of bacteria, and (*β* 1–6) glycosidic chains, mainly in the *β*-glucans of fungi [16]. In aqueous solution, *β*-glucans undergo conformational changes into triple helix, single helix, or random coil structures. The immune effects of *β*-glucans are thought to depend on their conformational complexity [17] and their anticancer potency is related to their molecular weight, degree of branching, and solubility in water [18].

The anticancer effects of *β*-glucans have been demonstrated primarily in* in vitro* and* in vivo* experimental systems but few studies in human are available [14]. *β*-Glucans have been used as adjuvant therapies in clinical trials, mainly in the Far East, with a positive effect on patient survival and quality of life [14]. Lentinan, extracted from Shiitake mushrooms, Krestin (PSK), a polysaccharide peptide extracted from* Trametes* (*Coriolus* or* Polyporus*)* versicolor*, and Schizophyllan (PolyC), from the fungus* Schizophyllum commune*, are available in Japan as dietary supplements and are indicated for the treatment of breast, digestive tract, and lung cancer (Krestin), for uterine cervical cancer (Schizophyllan), and for gastric cancer (Lentinan) [19].

Among the injection materials developed or currently used for vocal fold augmentation, none has anticancer effects [2, 3]. Thus, this novel hydrogel has the potential to be used in patients who have suspicious residual masses near the injection site to augment the paralyzed vocal fold without major concern for spreading the tumor through the needle pathway into the larynx. Moreover, subject to further validation in animal cancer models, this hydrogel has the potential to be used as an adjunct to conventional anticancer treatment regimens.

To control the biodegradation rate of hydrogels, cross-linking methods are used. Chemical cross-linking methods use chemicals to initiate the process. Free radicals, which are harmful to tissues, are produced by the decomposition of the initiator into fragments that attack the base polymer during the process. Initiation of chemical cross-linking is also limited by the concentration and purity of the initiators; moreover, there are problems associated with removing the initiator after cross-linking.

However, in the case of radiation processing, the radiation dose can be varied widely and is easily controlled. Due to the additive-free initiation and easy process control, radiation cross-linking techniques are suitable for the synthesis of hydrogels. Materials can be cross-linked with no added cross-linking agents. UV [20], electron beams (EB) [21], and *γ*-rays [22] are used frequently to induce the cross-linking of polymers. The energy of UV light is too low to penetrate into the deeper parts of the materials. Thus, cross-linking occurs on the surface instead of in the bulk of the material and the extent of cross-linking is limited [22]. High-energy EB is an effective energy source for radiation curing [23]; however, the penetration is not always sufficient for thick samples. In contrast, *γ*-rays can penetrate into materials to cross-link materials homogeneously [22]. Moreover, cross-linking and sterilization can be performed simultaneously. Thus, for the preparation of biomaterials, *γ*-radiation cross-linking has considerable advantages over other methods.

Inflammatory cell infiltration around the fatty tissue was greater in the nonirradiated *β*-glucan group than in the irradiated *β*-glucan group (Figure 5). A possible explanation is that cross-linking, by irradiation, prevented leakage of hydrogel into the surrounding tissue, and consequent recruitment of inflammatory cells. The mucosal wave amplitude was smaller on the injected side than on the noninjected vocal fold. We concluded that this result did not indicate the occurrence of adverse events, because we could identify no inflammatory reaction in the muscle, lamina propria, or epithelium of the injected vocal fold. It is possible that the mucosal wave amplitude of the injected vocal fold decreased due to a mass effect of the injected hydrogel.

The volume of the irradiated hydrogel in the vocal fold was maintained for 12 weeks of follow-up, without inducing complications such as migration from the injection site, inflammation, granuloma formation, or interference with vocal fold vibration due to viscoelastic mismatch. The volume of the injected hydrogel was not significantly reduced at 3 months after injection. However, examination did show a tendency for the volume to decrease slowly with time. Longer-term follow-up is needed for further evaluation of duration.

## 5. Conclusions

From the findings in the present study, we conclude that irradiated *β*-glucan hydrogel may provide a better vocal fold augmentation material for unilateral vocal fold palsy than nonirradiated hydrogel, because the irradiated hydrogel had slower degradation and excellent biocompatibility with the surrounding tissue.

## Supplementary Material

Supplementary Figure 1. β-glucan hydrogel maintained in Dulbecco's modified Eagle's medium (DMEM) containing 1 g L-1 glucose and 1% penicillin/streptomycin (PS) and then incubated at 37°C under CO2 conditions for 1 month. The volume of the irradiated hydrogel was maintained whereas the non-irradiated hydrogel dissolved completely.
Supplementary Figure 2. Examples of various β-glucan glycosidic linkages.
