# Supplementary material for: Vocal Fold Augmentation with Beta Glucan Hydrogel Cross-Linked by γ Irradiation for Enhanced Duration of Effect: In Vivo Animal Study
Source: Biomed Res Int. 2015 Dec 13;2015:592372. doi: 10.1155/2015/592372 (PMC4691524; doi:10.1155/2015/592372)
Supplement: Supplementary file 1 — Supplementary Figure 1. β-glucan hydrogel maintained in Dulbecco's modified Eagle's medium (DMEM) containing 1 g L-1 glucose and 1% penicillin/streptomycin (PS) and then incubated at 37°C under CO2 conditions for 1 month. The volume of the irradiated hydrogel was maintained whereas the non-irradiated hydrogel dissolved completely. Supplementary Figure 2. Examples of various β-glucan glycosidic linkages. [file 592372.f1.docx]

**Supplementary Figures**

Supplementary Figure 1. β-glucan hydrogel maintained in Dulbecco’s modified Eagle’s medium (DMEM) containing 1 g L^-1^ glucose and 1% penicillin/streptomycin (PS) and then incubated at 37°C under CO_2_ conditions for 1 month. The volume of the irradiated hydrogel was maintained whereas the non-irradiated hydrogel dissolved completely.


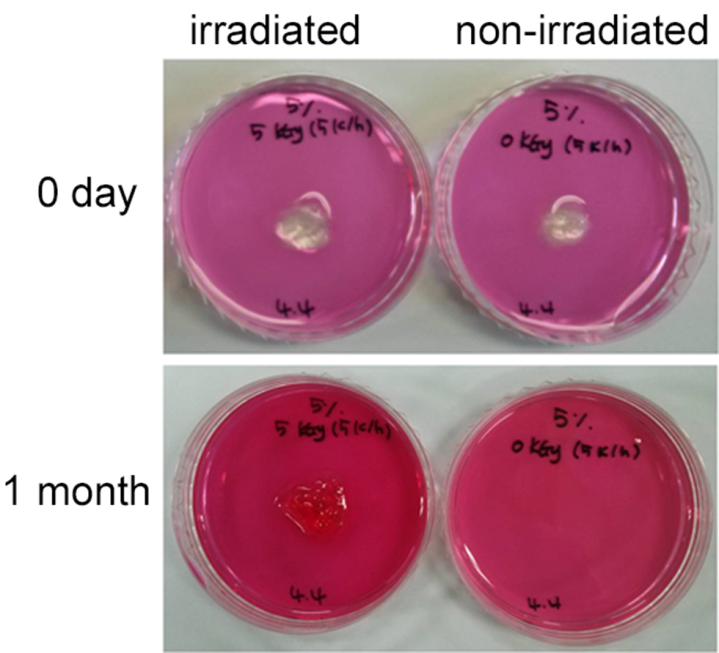


Supplementary Figure 2. Examples of various β-glucan glycosidic linkages.
